# Manganese dioxide- and cobalt oxide-doped hydroxyapatite with curcumin for orthopedic and dental applications

**DOI:** 10.1557/s43578-025-01575-x

**Published:** 2025-05-21

**Authors:** Joel Pilli, Gwenevere Gatto, Sameer Jain, Arjak Bhattacharjee

**Affiliations:** 1 Sustainable Manufacturing and Tissue Engineering Laboratory, Department of Materials and Metallurgical Engineering, New Mexico Institute of Mining and Technology, Socorro, NM 87801, USA; 2 Department of Biology, New Mexico Institute of Mining and Technology, Socorro, NM 87801, USA; 3 Department of Mechanical Engineering, New Mexico Institute of Mining and Technology, Socorro, NM 87801, USA

## Abstract

Due to their compositional similarities to bone, hydroxyapatite (HA)-based materials are used as bioactive ceramics for musculoskeletal repair. However, because of the lack of any inherent antibacterial properties, HA scaffolds have higher possibilities of post-surgical bacterial infections. The goal of this research is to fabricate alternate antibacterial and cytocompatible bone tissue engineering scaffolds using cobalt oxide (CoO)- and manganese dioxide (MnO_2_)-doped HA and plant-sourced curcumin from turmeric. Characterization results show no negative effects in phase and microstructure because of doping. When dopants and curcumin are combined, the antibacterial efficacy against *S. aureus* is ~ 95% after 24 h. The addition of dopants does not result in any cytotoxicity with the NIH3T3 cell line, and the bioactivity of this delivery system is confirmed in a physiological pH of 7.4. In summary, our findings provide an alternate method for manufacturing antibacterial scaffolds for orthopedic and dental applications employing curcumin-loaded CoO–MnO_2_-doped HA.

## Introduction

There is an increasing need for effective and novel solutions to bone regeneration and long-term stability of bone scaffolds due to the rise in cases of osteoporosis, bone cancer, various other types of bone diseases, and war injuries. These bone disorders lead to a significant economic liability worldwide [1]. The current gold standard of musculoskeletal reconstruction is with autologous bone graft, however, this needs multiple site surgeries and could lead to complications. An alternate strategy of tissue engineering is to replace the affected segment of bone with artificial scaffolds and grafts [2]. The goal of tissue engineering is to replace or reconstruct the affected body part with living materials or synthetic materials [3]. Given the increasing need for bone disorders and the limitations of traditional methods, the global tissue engineering market is expected to be ~ 36.34 billion by the year 2034 in which the highest market share was for orthopedics, musculoskeletal, and spine implants (~ 31% in 2023) [4]. The potential use of Hydroxyapatite [HA, Ca_10_(PO_4_)_6_(OH)_2_], for various bone tissue engineering applications has widely been studied by researchers [5–8]. HA has a similar composition and chemical structure to human bone, hence it is not rejected by the human body after implantation [9]. Despite the HA scaffold’s effectiveness in bone reconstruction, they lack inherent antibacterial properties and limited osteogenic potential without additional chemical modifications or growth factor addition [5, 6, 10]. Post-surgical infection and implant failure may occur when the HA scaffold’s surface has a bacterial infection, and this can further lead to more expensive, complicated, and painful revision surgeries [11]. Moreover, the increasing resistance to antibiotics and the challenge of providing site-targeted delivery complicates protection from bacterial strains and hinders postoperative care [12, 13].

One strategy for making HA antibacterial is to dope it with various cationic and anionic dopants. The crystal chemistry of HA allows tailoring its physical, mechanical, and biological properties with doping both in the cationic and anionic sites [14, 15]. Previous studies reported the assessment of the antibacterial properties of HA after doping it with various transition metals or anionic dopants [16, 17]. Many of these metals do not show any cytotoxic effects at low concentrations while still maintaining the antibacterial efficacy of doped HA [13, 18]. Moreover, these dopants not only boost HA’s biological performance but also improve its crystallinity and mechanical properties[18, 19]. Among these transition metals, the effects of cobalt and manganese doping are not yet well studied as compared to other transition metals such as zinc, magnesium, and silver. Limited available literature reports document that cobalt-substituted hydroxyapatite has shown an increase in compressive strength, measuring between 120 and 150 MPa, depending on the cobalt concentration. This is a significant increase compared to only hydroxyapatite [20]. Doping with various ternary divalent cations has a positive impact on improving the crystallinity and density of HA [21]. Cobalt (Co) is a component of many enzymes involved in the metabolism of bone. It is also an important component of vitamin B_12_ [22]. According to previous research, HA doped with cobalt ions (Co^2+^) provides antimicrobial properties against bacteria like *Staphylococcus aureus* (*S. aureus)* and *Escherichia coli* (*E. coli*) at implantation sites and also enhances the osteoblast proliferation and differentiation, which is crucial for bone development and reconstruction [23, 24].

The biological and mechanical properties of HA are affected when manganese (Mn^4+^) is added without reducing the material’s biocompatibility. Mn^4+^ addition is known to affect the bioceramics’ mechanical strength, phase composition, and sintering process—all of which are essential for successful implant integration [25, 26]. Biologically, Mn^4+^ is necessary for the synthesis of mucopolysaccharides, which are critical for cartilage formation and bone health. Mn^4+^ in HA enhances cell adhesion, proliferation, and viability, all of which are necessary for successful bone integration [27].

Curcumin is an active compound, that originates from the rhizome of turmeric (*Curcuma longa*). The curcumin molecule is hydrophobic meaning it cannot be dissolved in water. Various medicinal properties of curcumin such as antibacterial, anti-inflammatory, and chemo-preventive potential are well documented in available literature reports [28, 29]. Due to the natural medicinal chemicals being more readily available and have fewer adverse effects than synthetic medications, clinical research has recently shown a growing preference for them [30]. Despite this, there is a knowledge gap in the existing literature about the assessment of the biological properties of curcumin as a localized delivery vehicle specifically when directly incorporated onto CoO- and MnO_2_-doped HA. To address this, our study explored how cobalt oxide (CoO)- and manganese dioxide (MnO_2_)-doped HA combined with curcumin can be used as an localized antibacterial delivery system for orthopedic and dental applications. Cobalt oxide (CoO) and manganese dioxide (MnO_2_) are specifically chosen as dopants for HA in this study due to their intrinsic antibacterial properties and positive impacts on bone formation. The novelty of this research lies in the direct incorporation of plant-based derivative curcumin onto the doped HA scaffolds, which was then followed by an assessment of their biological characteristics. We have tested the scaffolds for their antibacterial effects, and found significant antibacterial efficacy against *S. aureus*. The results also show enhanced biological activity in the presence of these dopants and curcumin in a physiological pH of 7.4 with no negative effects from the doping process.

## Results

**Figure 1:**
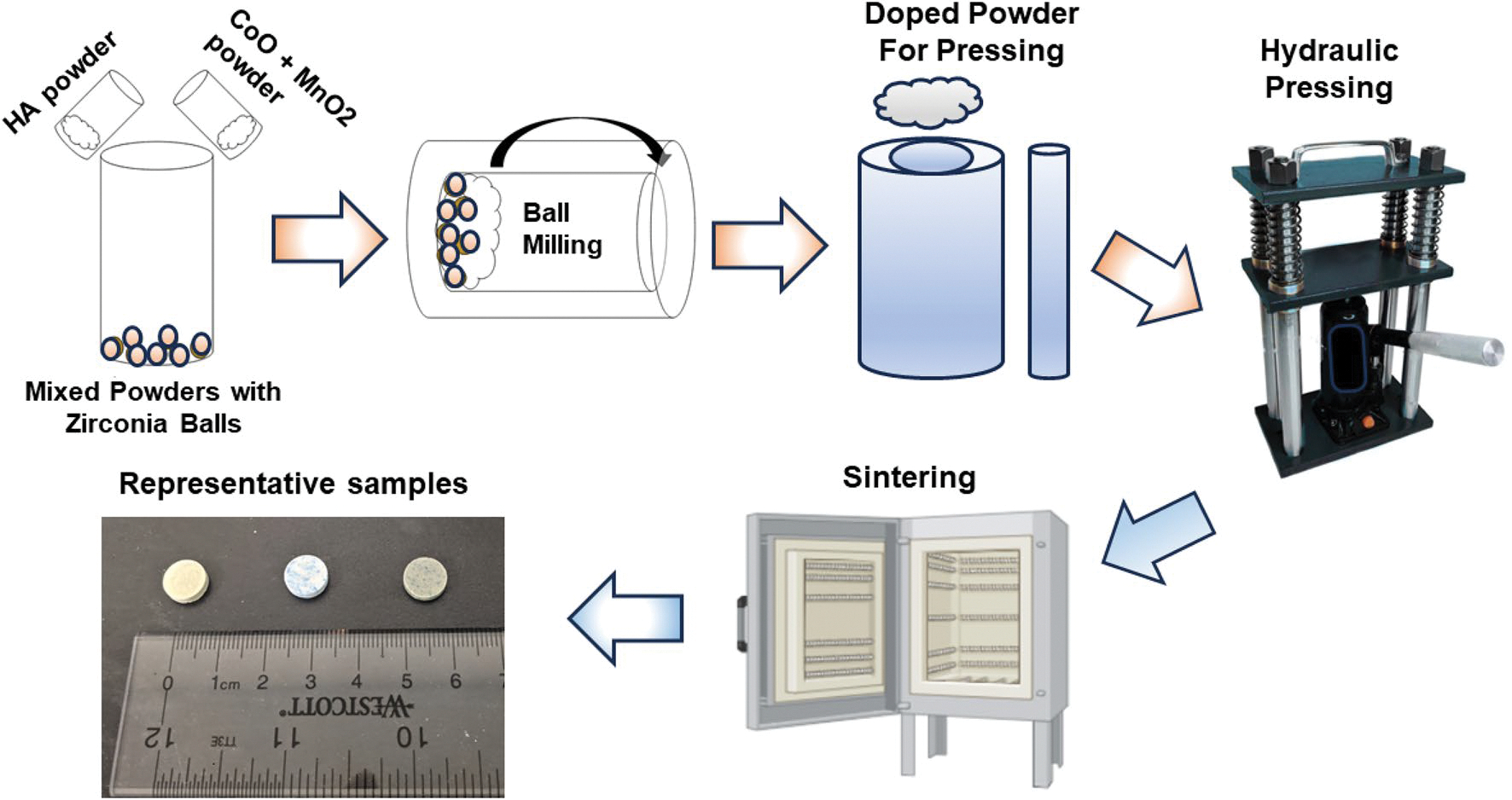
Sample preparation process schematic starting from doping HA with CoO, MnO_2_ followed by disc preparation with hydraulic pressing and sintering. Representative images of prepared discs are displayed.

The CoO and MnO_2_ powders were doped with HA via a ball milling process, pressed through a hydraulic pressing machine, and sintered, the sample production process and produced samples are displayed schematically in Fig. 1. Table 1 indicates the bulk density (g/cm^3^) alongside densification, volume, radial, and longitudinal shrinkage percentages post-sintering. The HA exhibits a bulk density of 2.63 ± 0.106 g/cm^3^. The cobalt-doped sample (CHA) shows a bulk density of 2.62 ± 0.052 g/cm^3^, while the manganese-doped (MHA) presents a higher bulk density of 2.71 ± 0.18 g/cm^3^. The CMHA composite shows a bulk density of 2.63 ± 0.06 g/cm^3^. In terms of densification shrinkage, HA at 83.91% ± 3.39, CHA at 83.53% ± 1.66, MHA at 86.3% ± 5.6, and the CMHA composite at 83.64% ± 1.83. The shrinkage measurements for HA are 57.99 ± 0.64% (volume), 25.44 ± 0.154% (radial), and 27.86 ± 3.48% (longitudinal). The CHA follows with 57.15 ± 1.57% (volume), 26.38 ± 0.162% (radial), and 21.69 ± 2.78% (longitudinal). MHA shows 61.9 ± 2.03% (volume), 26.87 ± 0.282% (radial), and 28.6 ± 3.95% (longitudinal), and the CMHA composite exhibits 57.59 ± 1.07% (volume), 25.69 ± 0.245% (radial), and 21.54 ± 2.18% (longitudinal).

**Figure 2:**
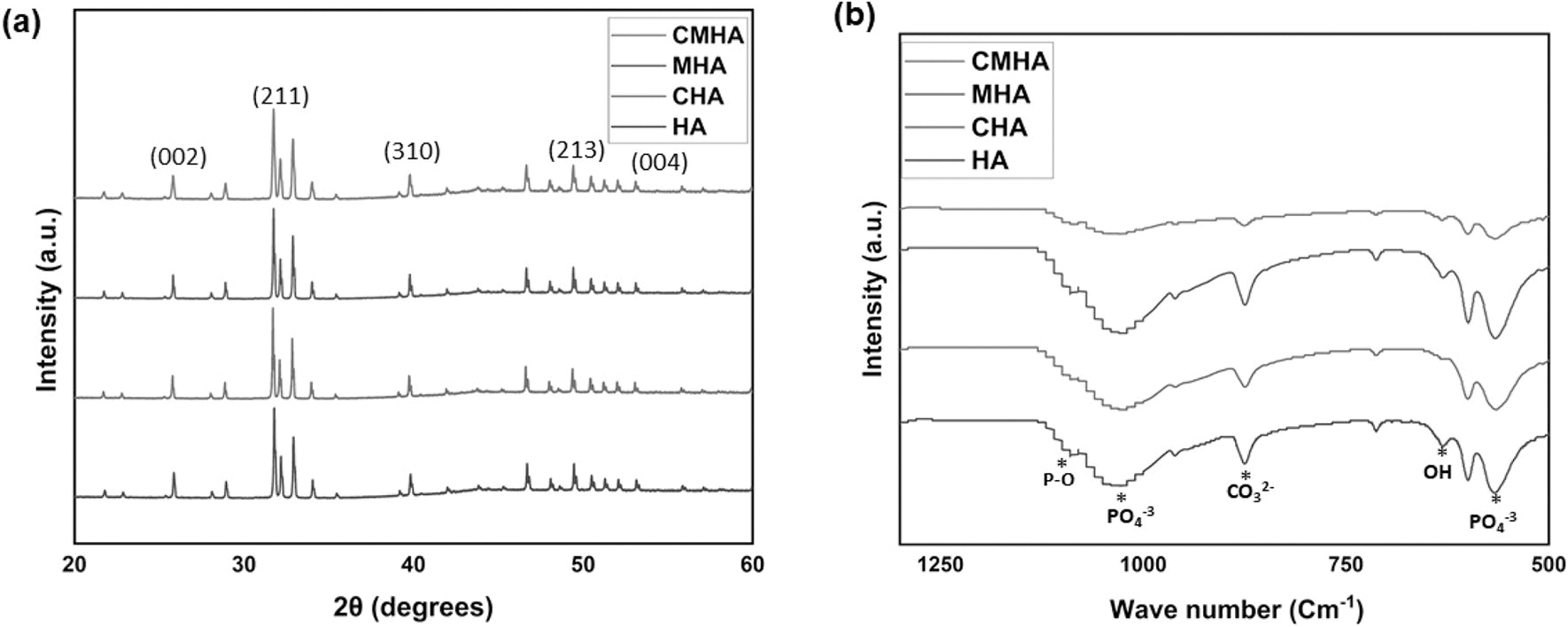
(a) XRD peaks of undoped HA, cobalt oxide-doped HA, manganese dioxide-doped HA, and cobalt oxide–manganese dioxide-doped HA in the range of 20–60°. The standard peaks of hydroxyapatite are observed both in the undoped and doped samples, (b) the FTIR spectra of the aforementioned compositions in the range 500–1300 cm^−1^ indicate corresponding functional groups to hydroxyapatite. No adverse effects in the functional groups are noticed as a result of doping.

**Figure 3:**
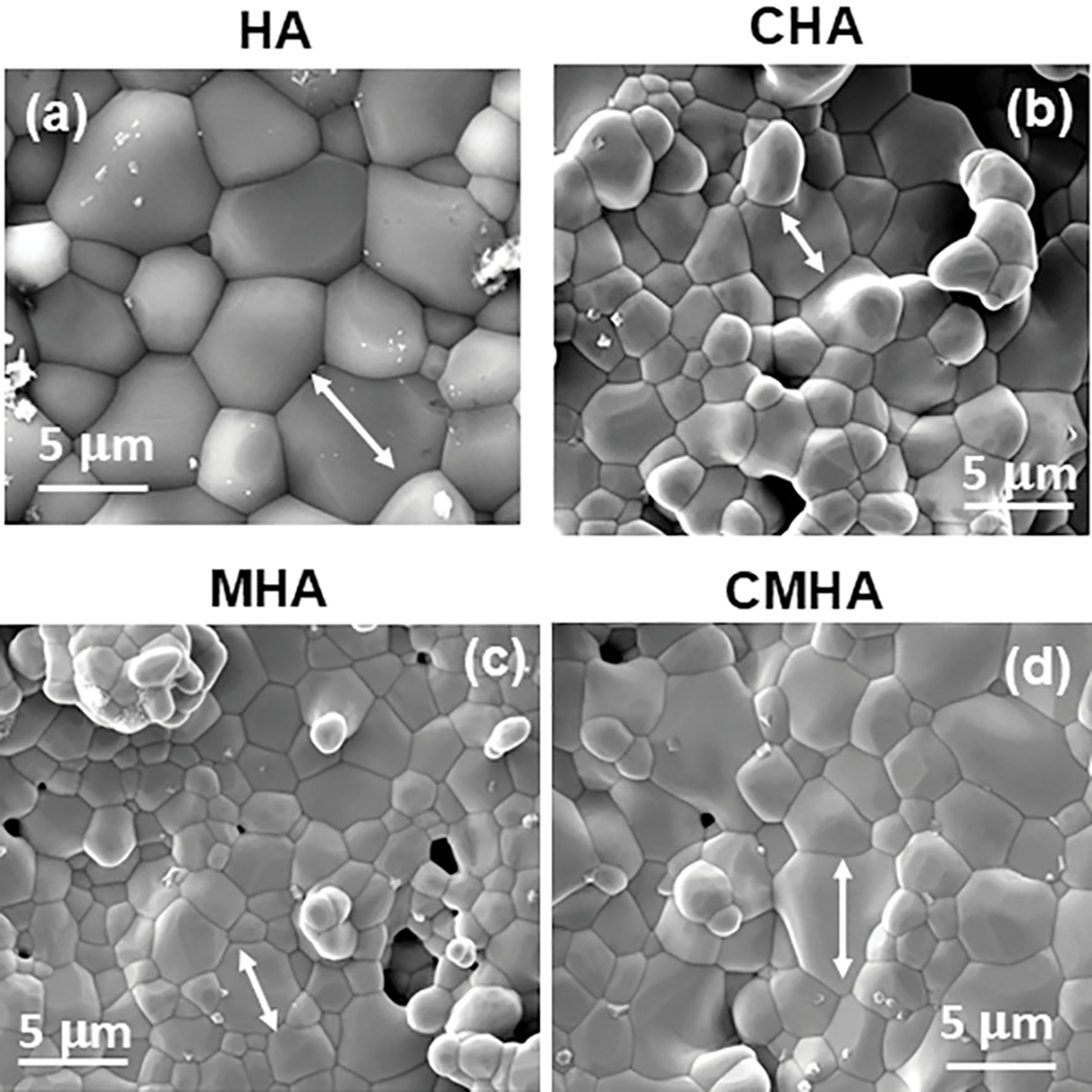
The FESEM images displaying the morphology of the sintered samples (a) Undoped HA, (b) HA doped with CoO, (c) HA doped with MnO_2_, and (d) HA co-doped with CoO and MnO_2_.

Figure 2(a) displays the X-ray diffraction (XRD) results of the HA and sintered HA samples, in a range of 20–60°. The tested samples were HA, HA doped with Cobalt oxide (CHA), HA doped with Manganese dioxide (MHA), and the composite sample with both cobalt oxide and manganese dioxide doping (CMHA). The HA phase is confirmed from JCPDS #09–0432 [1]. The characteristic peaks of HA samples were observed and have been annotated in the figure. Doping HA with CoO and MnO_2_ doesn’t cause any adverse effects on the phase formation post-sintering. No significant presence of any secondary phase in noticed in the XRD spectra. In Fig. 2(b), the FTIR spectra of undoped and doped HA variants (HA, CHA, MHA, CMHA) show the vibrational modes and corresponding functional groups in the 1300–500 cm^−1^ range. The spectra of all show an absorption band at ~ 1100 cm^−1^ and 600 cm^−1^, denoting the presence of phosphate groups (PO_4_^3−^), and hydroxyl groups (OH) at around 630 cm^−1^ [31]. Doping HA with CoO and MnO_2_ does not lead to any adverse effect with any functional groups. Based on Fig. 3(a)–(d), all the sintered samples have clearly defined grain boundaries suggesting successful sintering of the green compact [32, 33]. A similar morphology is noticed across all tested compositions.

**Figure 4:**
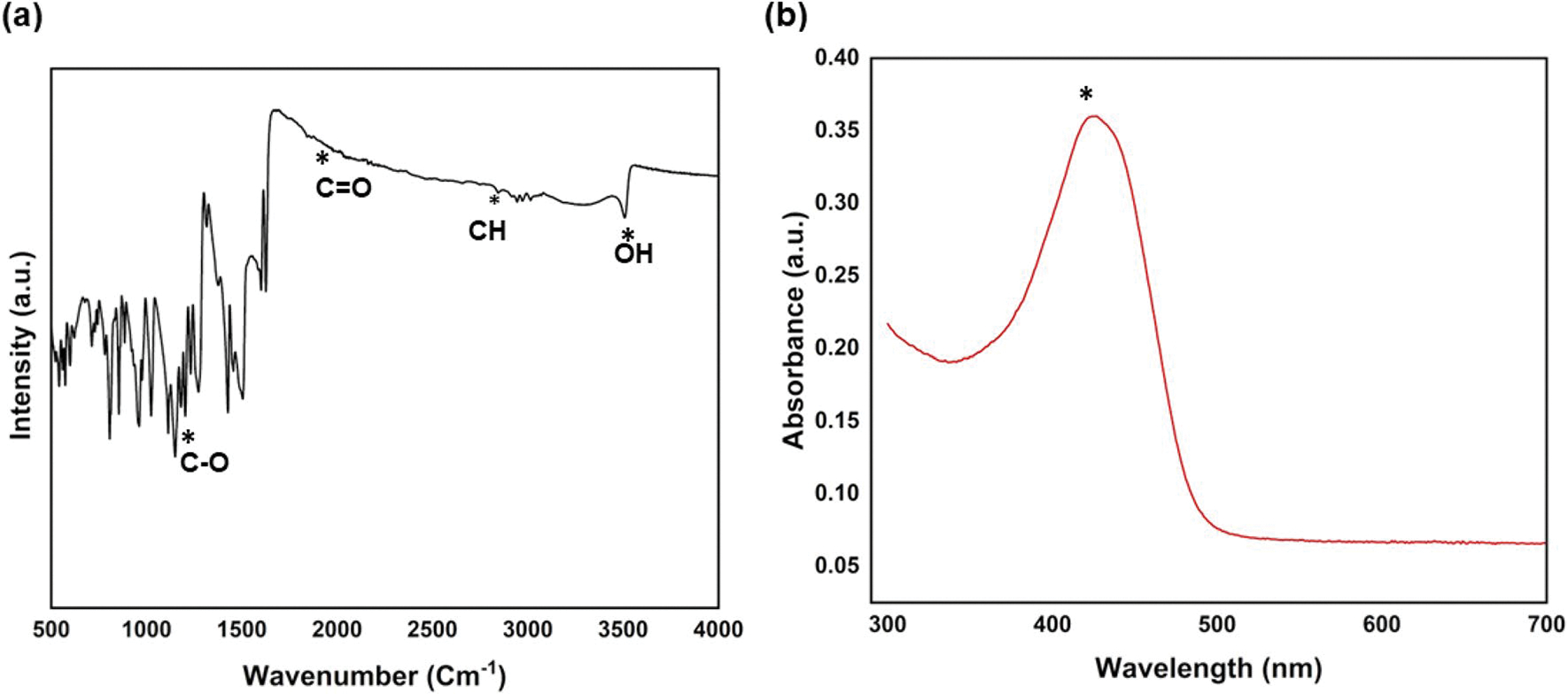
(a) FTIR spectra of curcumin show corresponding functional groups, (b) the UV–VIS spectra show the signature peak of curcumin at ~ 425 nm.

The FTIR spectrum of curcumin shown in Fig. 4(a) exhibits a significant peak at around 3500 cm^−1^, representing the O–H stretch. The stretch at ~ 1670 cm^−1^ indicates the C = O stretch. The vibrations near 1550 cm^−1^, represent the C = C vibrations, and a bending vibration near 1280 cm^−1^, indicates the C–O vibrations. These obtained FTIR peaks are consistent with previous works [34–36]. In the UV–Vis spectrum of curcumin shown in Fig. 4(b), a prominent absorption peak is noticed at ~ 425 nm. This peak is the corresponding signature peak of curcumin. Previous studies also reported similar observations [37].

**Figure 5:**
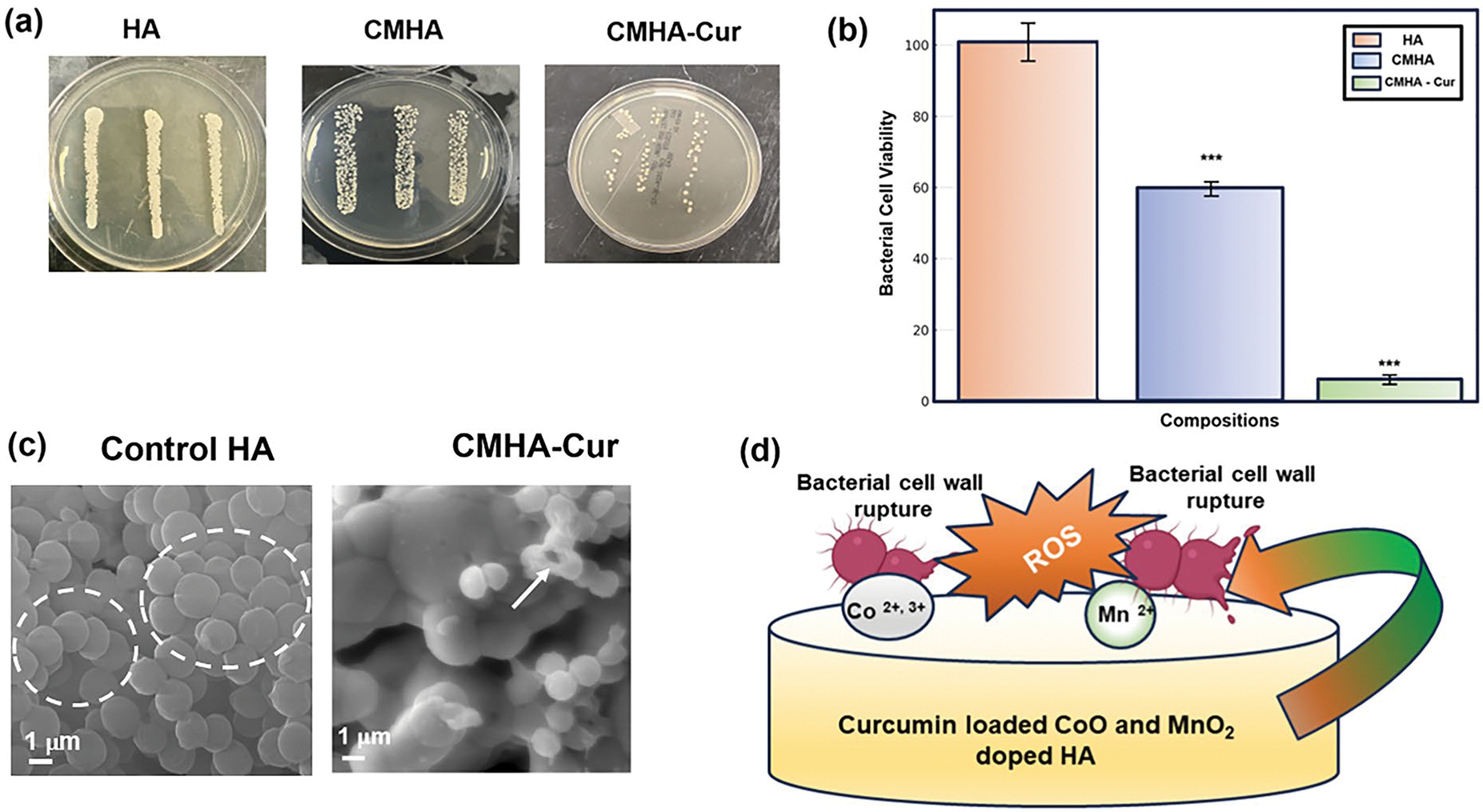
Antibacterial efficacy based on the modified ISO 22196: 2011 standards at 24 h-time point, after the interaction between the samples and bacteria, (a) prominent bacterial colony growth in the HA sample is noticed, whereas the sample doped with CoO-MnO_2_ shown notable reduction in bacterial colonies and CoO–MnO_2_ loaded with curcumin shows a markedly reduced presence of bacterial colonies on the agar plates, (b) bacterial cell viability quantification reveals that the CMHA composition achieves approximately ~ 40% antibacterial effectiveness and CMHA-Cur composition achieves approximately ~ 95% antibacterial effectiveness post 24 h of interaction with the bacteria (***P < 0.0001), (c) the FESEM images indicate denser bacterial population on the control HA sample compared to the significant reduction seen in the treatment CMHA-Cur sample. The dotted circle in the control sample indicates dense bacterial colonies and the arrow in the treatment sample indicates a ruptured bacterial cell wall in the presence of the antibacterial components, (d) a schematic diagram illustrating the antibacterial action due to Curcumin, CoO, and MnO_2_ is presented.

**Figure 6:**
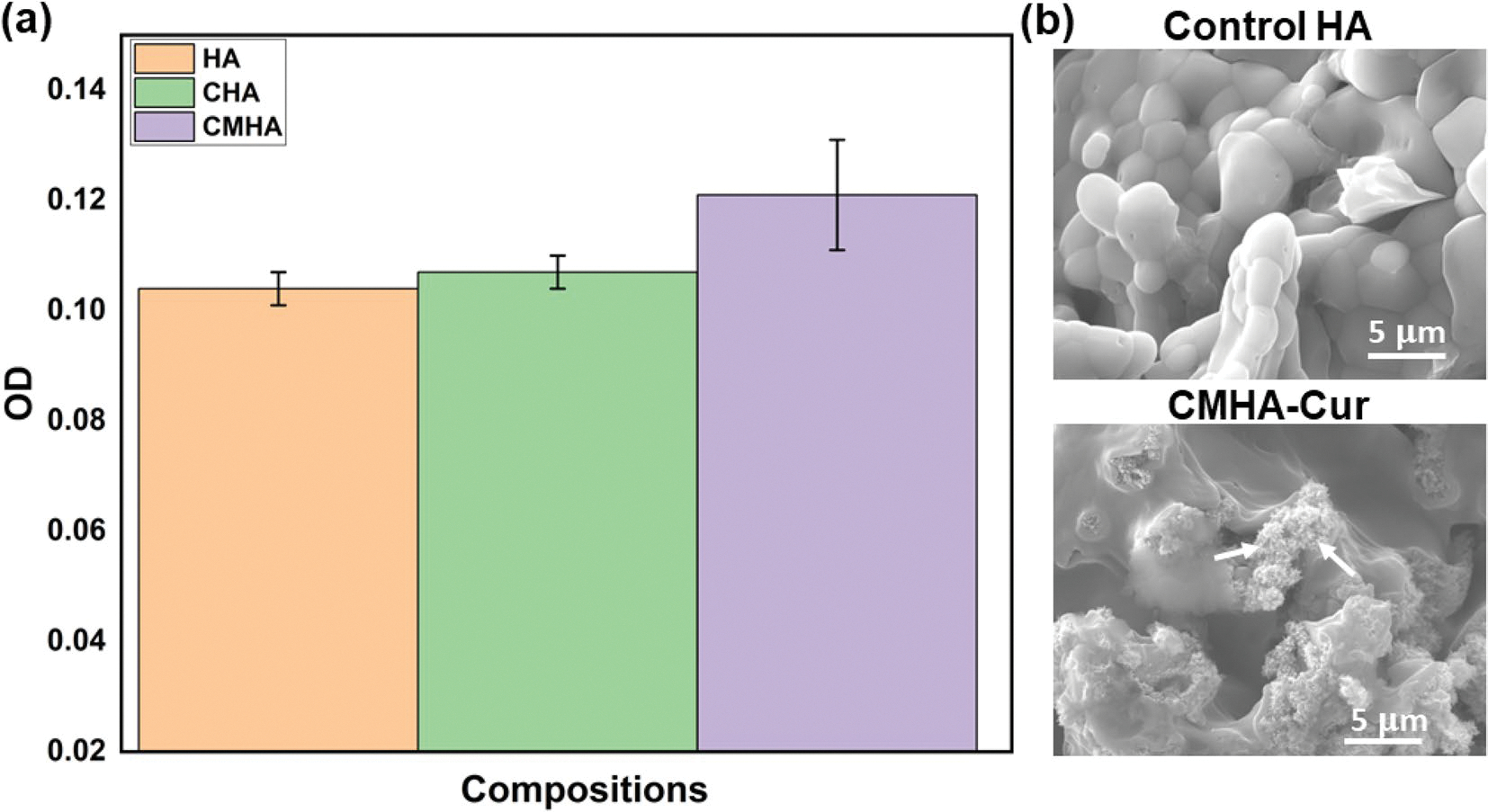
(a) The MTT assay after 24 h of sample-cell interaction shows no cytotoxic effects across all compositions, (b) the bioactivity results show no significant apatite layer formation on the control HA sample, whereas the treatment CMHA-Cur sample shows initial formation of apatite layer, which is marked with arrows.

The antibacterial efficacy results of tested samples against *S. aureus* are shown in Fig. 5(a), (b). The agar plate results [Fig. 5(a)] show denser bacterial colonies in the control HA sample, the cobalt oxide (CoO)- and manganese dioxide (MnO_2_)-doped hydroxyapatite (CMHA) scaffold have shown a notable reduction in the bacterial colonies compared to control HA sample and while the treatment sample CMHA-Cur having significantly smaller number of bacterial colonies. Quantitative measurement of bacterial colonies [Fig. 5(b)] shows that the treated sample (CMHA – Cur) has ~ 95% antibacterial efficiency and the CMHA scaffold shown significant antibacterial properties, achieving ~ 40% antibacterial efficacy against *S. aureus* when compared to the control HA scaffold. Additionally, the FESEM images [Fig. 5(c)] obtained after bacterial culture show a higher density of bacterial colonies in the control HA, which was reduced due to the presence of antibacterial CoO, MnO_2_, and curcumin. The dotted circle in the FESEM figure of the control HA sample marks the bacterial colonies, and the arrow in the FESEM of the CMHA-Cur sample shows a ruptured bacterial cell wall along with an overall reduction in bacterial density than the control. The proposed antibacterial mechanism due to reactive oxygen species (ROS) generation is shown in Fig. 5(d). Figure 6(a) shows the cytotoxicity results of the tested samples with the NIH3T3 cell line. The obtained results show that no composition is cytotoxic as per the ISO 10993 standard [31]. The cell viability is similar across all tested compositions without any significant show any significant flaky apatite layer formation in contrast, difference after 24-h of sample-cell interaction. The FESEM the CMHA-Cur sample shows initial flaky apatite formation results of the selected samples after bioactivity testing are at an early stage of 1 week. Noticed the flaky apatite layer is shown in Fig. 6(b). Both the tested samples show stability in marked with arrows. the physiological pH of 7.4. The control HA samples do not show any significant flaky apatite layer formation in contrast, the CMHA-Cur sample shows initial flaky apatite formation at an early stage of 1 week. Noticed the flaky apatite layer is marked with arrows.

## Discussions

### Effects of manganese dioxide and cobalt oxide doping on HA

Previous works report that the theoretical density of hydroxyapatite is ~ 3.16 g/cm^3^ [38]. In our study, the bulk density of the doped samples indicates a stable lattice structure even with the addition of dopants with no adverse effects. All the sintered samples exhibit densification shrinkage percentages between approximately 83% and 86% after conventional sintering. This high level of densification indicates effective consolidation and reduction of porosity, which is crucial for achieving the mechanical strength required in bone and dental implants. Previous studies on the densification of HA or doped HA by conventional sintering indicate a similar sintered density for the studied samples [39]. The MnO_2_ addition can lead to full densification at lower temperatures, showing the dopant’s effectiveness in improving the sintering process and enhancing the mechanical properties of the ceramic matrix [40]. This aligns with the increased densification shrinkage and slightly increased average bulk density observed in MHA, suggesting that MnO_2_ not only aids in densification but might also contribute to providing structural stability necessary for biomedical implants.

### Influence of doping on physical properties, microstructure, and cytotoxicity

The XRD and FTIR results shown in Fig. 2(a) (XRD) and Fig. 2(b) (FTIR), suggest that doping HA with CoO and MnO_2_ does not cause any adverse results in the phase formation of HA, and the results show that they were well incorporated into the HA lattice without significantly changing its crystallographic structure. This observation has been reported in previous studies as well [41–43]. The crystallite size generally decreases with the substitution of metal ions (Mn^4+^, and Co^2+^) in hydroxyapatite (HA), which is consistent with previous studies’ findings [44]. The ionic radii of Ca^2+^ is ~ 1.00 Å, whereas the ionic radii of Co^2+^ is ~ 0.74 Å and that of Mn^4+^ is ~ 0.53 Å. Hence, the substitution of HA with these cations leads to a reduction in unit cell volume. Additionally, these cations have a positive influence on new bone formation, introducing antibacterial properties, etc. A lower concentration of dopants does not adversely affect the cell viability. Due to these reasons, these specific dopants at a lower concentration are selected for the study.

The FESEM images [Fig. 3(a)–(d)] show that the doped and undoped samples have a similar grain shape and morphology, with all showing distinct grain boundaries confirming full material sintering. In Table 1, a similar trend is noticed in the densification results for both doped and undoped samples. This finding is consistent with recent research on the densification of HA and doped HA using conventional sintering, which reported identical densities for the samples studied [39]. Our study’s FESEM microstructures [Fig. 3(a)–(d)] provide additional support for these findings. To ensure that the incorporation of these transition metals is not happening at the cost of cytotoxicity, we performed cytotoxicity assessment with NIH3T3 cell lines. The obtained cell viability results (Fig. 6a) show that the incorporation of dopants does not cause any toxic effects on the cells. The ISO 10993 criteria for non-cytotoxicity is fulfilled with these doped compositions. Following this, curcumin was loaded onto the doped samples and evaluated for antibacterial activity and bioactivity.

### Curcumin as an alternate antibacterial agent

The search for effective antibacterial agents derived from natural sources has been of significant importance within the clinical research field. Ayurveda, the ancient traditional Indian medical literature well documented the clinical disorders treatments using various types of natural medicinal compounds [45]. Curcumin, the active component of turmeric (*Curcuma longa*) rhizome is a polyphenolic compound, known for its broad spectrum of therapeutic properties, including its antibacterial activity. Unlike conventional antibiotics, curcumin offers the advantage of disrupting bacterial cell walls and inhibiting cell division without promoting bacterial resistance against antibiotics, making it an attractive candidate for long-term therapeutic use [46]. This work’s novelty lies in the investigation of the potential to use curcumin as a localized delivery vehicle after direct incorporation into CoO and MnO_2_-doped HA. The localized delivery of curcumin is specifically helpful in bone tissue engineering as it allows for site-specific therapeutic action, reducing systemic toxicity and minimizing the risk of off-target effects. The localized drug delivery ensures a high concentration of the antibacterial agent directly at the infection site, which is important for overcoming biofilm-associated resistance and promoting healing. Furthermore, curcumin’s natural ability to release in a controlled and sustained manner enhances its efficacy in overcoming bacterial infections [47]. In addition to its antibacterial properties, curcumin has been shown to modulate the osteo-immune microenvironment by promoting the production of osteogenesis-related cytokines such as bone morphogenetic protein-2 (BMP-2) and transforming growth factor-beta (TGF-β), both of which are critical for bone tissue regeneration. Curcumin has also been shown to upregulate vascular endothelial growth factor (VEGF), therefore improving nutrient delivery during bone repair. These positive effects of promoting new bone formation makes curcumin a promising agent for various bone tissue engineering applications [48, 49]. The FTIR analysis for curcumin, represented in Fig. 4(a), showed prominent peaks that correspond to O–H, C = O, C = C, and C–O bonds. These spectral features align with findings from prior research. In Fig. 4(b), curcumin’s main absorption peak was observed at ~ 425 nm. These results and absorption peaks align with the other observations in previous studies [34–36]. Curcumin has been widely explored for its several therapeutic benefits. Curcumin has shown significant anti-inflammatory and antioxidant properties, which contribute to its potential in treating various chronic diseases such as arthritis, metabolic syndrome, and other inflammatory conditions [50, 51]. Previous studies have additionally shown curcumin’s potential in neurodegenerative and cardiovascular diseases, as well as its chemopreventive properties, where it has demonstrated efficacy in influencing multiple cellular processes that are linked to the progression and survival of cancer cells [52]. Additionally, curcumin has been found to have beneficial effects in managing conditions related to oxidative stress and inflammation, which are common in metabolic disorders [53]. Our objective is to use curcumin’s therapeutic potential for bone tissue engineering applications.

### Antimicrobial effects and bioactivity

Our study focuses on the antibacterial properties of curcumin-loaded CoO and MnO_2-_doped hydroxyapatite (HA) against the osteomyelitis-causing *S. aureus*. The current treatment approach to osteomyelitis is surgical intervention along with prolonged high-dose antibiotic consumption. However, uncontrolled antibiotic use has resulted in multiple drug-resistant bacteria, which is very difficult to treat. One recent statistics show that ~ 10 million annual deaths are predicted by 2050 due to drug-resistant bacteria if this situation is not controlled now [54]. Curcumin’s antibacterial potential is still untapped from a clinical perspective and curcumin resistance bacteria has not yet been discovered. Our approach is to use curcumin’s advantages over conventional antibiotics in combination with antibacterial dopants for bone tissue engineering applications. Figure 5(a), (b) show that dopants alone achieve an antibacterial efficacy of approximately 40%. However, when combined these dopants with curcumin the antibacterial efficacy significantly increases, reaching up to ~ 95%. Curcumin ruptures the bacterial cell wall and inhibits the formation of bacterial virulence factors and biofilm growth, both of which are necessary for the bacteria’s survival and proliferation. This activity is enhanced by the inducing of oxidative stress within the bacterial cells [46]. The dopants utilized, CoO and MnO_2_, have substantial antibacterial properties. CoO, similar to other transition metals like ZnO, creates reactive oxygen species (ROS), which are the major mechanism for its bactericidal action, efficiently killing bacteria and decreasing their adherence to surfaces with Co^2+^ ions. These ions reduce bacterial cell membrane permeability and interfere with amino acid metabolism. Furthermore, Co^2+^ interacts with important groups in proteins, nucleic acids, and biological enzymes, limiting bacterial growth [55]. Mn^4+^ ions have demonstrated antibacterial effectiveness as well, particularly in synthesized complexes with other antibacterial agents like curcumin. Similarly, MnO_2_ also improves antibacterial properties by producing ROS and establishing an alkaline environment around the implant, therefore limiting bacterial growth and adherence. Interaction with manganese-containing surfaces produces a proton electrochemical gradient, interrupting ATP production and causing bacterial death. This combination has shown enhanced efficacy against a range of bacterial strains, suggesting that manganese can support curcumin’s antibacterial action [56, 57]. These processes and findings demonstrate CoO and MnO_2_’s ability, when coupled with curcumin, to successfully battle *S. aureus*-related osteomyelitis infections. The FESEM results [Fig. 5(c)] from our study show that combining curcumin with CoO-MnO_2_ dopants leads to a significant reduction in bacterial colonies compared to the control. The suggested antibacterial mechanism due to ROS generation is shown in Fig. 5(d). Bioactivity of scaffolds plays an important role in early stage bone regeneration after implantation [58]. Our obtained results of initial bioactivity studies show that the presence of curcumin and dopants with HA leads to enhanced bioactivity than the control HA scaffold surface. Initial flaky apatite layer formation is visible in the FESEM image of the CMHA-Cur sample surface and marked with arrows [Fig. 6(b)]. Additionally, the scaffolds maintain their stability in a physiological pH of 7.4, which is a primary criterion for any scaffolds to investigate their therapeutic potential for bone tissue engineering. This system provides potent antibacterial effects while also aligns with the principles of personalized medicine by addressing site-specific therapeutic needs. The ability to deliver curcumin directly to the affected area allows optimal bioactivity, reduced systemic side effects, and improves patient outcomes, showing its potential to transform current approaches in bone regeneration [59].

### Contributions to science and future direction

The major contribution to the science of this work is the assessment of biological properties of CoO–MnO_2_-doped HA after the direct incorporation of curcumin as an alternate and novel antibacterial scaffold for bone tissue engineering. Future studies could be directed towards assessing the long-term in vitro efficacy of this designed localized delivery vehicle, followed by the assessment of in vivo therapeutic potential. The graphical abstract of our work summarizes the key findings of our work.

## Conclusions

Our research has demonstrated the successful fabrication of HA and doped CoO–MnO_2_ -HA scaffolds using in-house uniaxial pressing, followed by sintering and an assessment of their microstructure and physical properties. Phase characterization with XRD and FTIR, as well as microstructural study with FESEM, demonstrate that co-doping HA with CoO and MnO_2_ has no adverse effects on phase formation or microstructure. The successful densification of the produced samples is confirmed by density and shrinkage measurements after sintering. The Curcumin, used as a novel antibacterial agent through direct inclusion on the scaffold surface, has demonstrated ~ 95% antibacterial activity against osteomyelitis causing *S. aureus*, which was attributed to the combined effects of curcumin and dopants. Additionally, the CoO and MnO_2_-doped HA scaffolds (CMHA) alone demonstrate ~ 40% antibacterial efficacy, showing the antibacterial effectiveness of the dopants. This antibacterial effect of dopants has been significantly enhanced by the addition of curcumin, which disrupts bacterial cell walls and inhibits biofilm formation without inducing resistance. Importantly, cytotoxicity assessments confirm the biocompatibility of the doped scaffolds, fulfilling ISO 10993 criteria. In conclusion, the co-doped CoO and MnO_2_ HA scaffolds with curcumin has a combined effect that enhances the antibacterial properties, bioactivity and mechanical stability. These results suggest that the scaffolds have significant potential to be used as an alternate scaffold for a variety of orthopedic and dental applications.

## Materials and methods

### Sample fabrication

The commercial HA powder (NEI, USA) was doped with manganese dioxide (MnO_2_) and cobalt oxide (CoO) at concentrations of 0.25 wt% and 0.35 wt%, respectively, utilizing a two-hour ball milling process at 80 rpm and a 1:2 powder-to-ball ratio [17]. Uniaxially pressurized discs were prepared using doped and undoped powders in a hydraulic press for 2 minutes. Sintering at 1250 °C for 2 h was then carried out, as previously mentioned [60]. Moving forward in the manuscript, the undoped hydroxyapatite will be identified as HA, the hydroxyapatite doped with CoO will be called CHA, the hydroxyapatite doped with MnO_2_ will be called MHA, and for the rest of the study, CoO and MnO_2_ doped HA will be referred to as CMHA.

### Measurement of densification and dimensional shrinkage

The geometrical dimensions of the samples were measured to calculate the bulk density. The ratio between the samples’ bulk density and theoretical density was used to calculate the densification following sintering. By measuring the dimension change for volume following sintering with respect to the volume of the green compacts, the volume shrinkage was computed. Similar measurements of the diameter change, and height change before and after sintering were made for the radial and longitudinal shrinkage (*n* = 3). The average and standard deviation of the results (%) are shown in Table 1.

### Phase and microstructural investigation

The phase characterization was performed with x-ray diffraction (XRD) at 45 kV and 40 mA, using a Cu–K*α* radiation of 1.54 Å, in a PANalytical Empyrean apparatus [20° ≤ 2*θ* ≤ 60°, step size: 0.015° (400 s/step)].

Using Fourier Transform Infrared Spectroscopy (FTIR) (Thermo Scientific IS50-FT-IT), the functional groups of HA, MHA, CHA, and CMHA were characterized in the 500–2000 cm^−1^ range. Utilizing Field Emission Scanning Electron Microscopy (FESEM) at a voltage of 15 kV, the effects of CoO and MnO_2_ doping on the morphology and microstructure of HA were examined. To make the samples conductive, a platinum layer was added before SEM imaging.

### Curcumin loading

A concentration of 5 mg/mL of curcumin (98.0% Millipore Sigma, St. Louis, USA) was dissolved in pure ethanol. After pipetting this solution, an appropriate amount of curcumin (500 μg) was loaded onto each scaffold, followed by the assessment of biological properties. The curcumin loading approach is described in a previous work [17].

### Assessment of biological properties

#### Antibacterial efficacy testing (in accordance with ISO 22196: 2011 standards)

The antibacterial activity against *S. aureus*, a microbe obtained from Carolina Biological Supply Company in Burlington, NC, was measured. Using a UV–Vis spectroscopy microplate reader (manufactured by Biotek), measurements of the optical densities of bacterial suspensions at various concentrations were made at 625 nm and compared with the McFarland standard [61]. After the samples were sterilized, 10^5^ CFU of bacteria were added to each sample inside the 24-well plates, and 1 mL of broth media was added. Following a 24 h incubation period at 37 °C, the samples were placed in glass vials, combined with 1 mL of phosphate buffer solution (PBS), vortexed for 15 s, and then plated in 10 μL increments on agar plates using streaking. After a 24-h incubation period at 37 °C, the streaked plates were examined and counted after taking photographs.

#### Morphological characterization

The field-emission scanning electron microscope (FESEM) was utilized to study and evaluate the morphology of the bacteria after the interaction of bacterial loading with each sample for 24 h. After fixing the sample in 0.1 M PBS with 2% paraformaldehyde and 2% glutaraldehyde, it was stored at 4 °C overnight. Following rinsing with 0.1 M PBS, each sample underwent multiple concentrations of ethanolic dehydration (30, 50%, 70%, 95%, and 100% three times). After hexamethyldisilane (HMDS) drying for overnight inside a fume hood, the sample surface was coated in platinum using a platinum coater.

### Assessment of cytocompatibility

#### Cell seeding on sample surfaces

As mentioned in our earlier study, the NIH3T3 cell line was utilized to perform the cytocompatibility test following CoO and MnO_2_ doping. The medium used for this experiment was Dulbecco’s Modified Eagle’s Medium (DMEM), combined with 10% v/v fetal bovine serum (FBS) and 1% v/v penicillin/streptomycin [62]. A cellular seeding density of ~ 20,000–25,000 per sample was used per sample followed by adding 1 mL culture media to each well of the 24-well plate. After this, the well plate incubation was done at 37 °C in a 5% CO_2_ environment.

#### MTT assay for cytotoxicity assessment

To investigate cytotoxicity, MTT (3-(4,5-dimethylthiazol- 2-yl)-2,5-diphenyl tetrazolium bromide) assay was used after cell-material interactions. After removing the culture media from each sample, 100 μL of MTT solution was poured into each well, followed by the addition of 900 μL of media. Then the well plates were kept inside an incubator for 2 h at 37 °C. In the next step, 600 μL of MTT solubilizer was added to dissolve the purple formazan crystals and 100 μL of that resultant solution in multiple replicates was transferred to a 96-well plate to measure the optical density using UV–VIS spectroscopy. The cell viability was quantified from the obtained results. Multiple replicates were used throughout the whole study followed by statistical analysis of the obtained results.

### Assessment of bioactivity at physiological pH

The bioactivity of the grafts was studied in PBS at a physiological pH of 7.4. The grafts were submerged in 4 mL of PBS at 37 °C for a week, with the PBS solution changed every two days. After 1 week, the dried samples were examined using FESEM to determine bioactivity. The samples were coated with platinum before FESEM.

## Figures and Tables

**TABLE 1:** The bulk density (g/cm^3^), shrinkage after densification, and also the volume, radial, and longitudinal shrinkage (%) post-sintering.

Sample ID	Bulk density (g/cm^3^)	Densification Shrinkage (%)	Volume shrinkage (%)	Radial shrinkage (%)	Longitudinal shrinkage (%)

HA	2.63 ± 0.106	83.91 ± 3.39	57.99 ± 0.64	25.44 ± 0.154	27.86 ± 3.48
MHA	2.71 ± 0.176	86.3 ± 5.6	61.90 ± 2.03	26.87 ± 0.282	28.60 ± 3.95
CHA	2.62 ± 0.052	83.53 ± 1.66	57.15 ± 1.57	26.38 ± 0.162	21.69 ± 2.78
CMHA	2.627 ± 0.06	83.64 ± 1.83	57.59 ± 1.07	25.69 ± 0.245	21.54 ± 2.18

It could be seen that all samples exhibit a similar range of bulk density and densification (~ 83% to ~ 86%) post-sintering, indicating a consistent level of porosity reduction across different dopant conditions.

## Data Availability

Data presented in this paper will be made available upon reasonable request to the corresponding author. The experimental data collected and its analysis are available from the corresponding authors on reasonable request.
